# Identification of shell-color-related microRNAs in the Manila clam *Ruditapes philippinarum* using high-throughput sequencing of small RNA transcriptomes

**DOI:** 10.1038/s41598-021-86727-9

**Published:** 2021-04-13

**Authors:** Jianfeng Ding, Qiang Wen, Zhongming Huo, Hongtao Nie, Yanjie Qin, Xiwu Yan

**Affiliations:** 1 Dalian Ocean University, Dalian, 116023 China; 2 Engineering Research Center of Shellfish Culture and Breeding in Liaoning Province, Dalian, 116023 China

**Keywords:** Evolution, Molecular biology

## Abstract

Shell-color polymorphism is a common phenomenon in several mollusk species and has been associated with thermal capacity, developmental stability, shell strength, and immunity. Shell-color polymorphism has been related to the differential expression of genes in several signal transduction pathways; however, the functions of micro-RNAs (miRNAs) in shell-color formation remain unclear. In the present study, we compared high-quality, small-RNA transcriptomes in three strains of the Manila clam *Ruditapes philippinarum* with specific shell-color patterns, artificially selected for six generations. Totals of 114 known and 208 novel miRNAs were identified by high-throughput sequencing, of which nine known and one novel miRNA were verified by stem-loop quantitative real time-polymerase chain reaction. Predicted miRNA targets were subjected to Gene Ontology and Kyoto Encyclopedia of Genes and Genomes pathway enrichment analyses. miR-137 and miR-216b and the Hedgehog signaling pathway and Wnt signaling pathway were identified as being potentially involved in pigment formation and regulation in *R. philippinarum*. These results may help to clarify the role of miRNAs in shell coloration and shed light on the mechanisms regulating color formation in bivalve shells.

## Introduction

Shell-color polymorphism is a common phenomenon among mollusks. Shell pigmentation has been potentially associated with various factors, including predation^1^, climatic effects^2,3^, thermal capacity^2,4,5^, developmental stability^6^, shell strength^7^, and immunity^8,9^. Mollusk shells are multi-layered structures consisting of calcium carbonate crystals, together with proteinaceous material and pigments^10^. The shell is generated by the outer fold of the mantle, and shell growth and pigmentation are regulated by neurosecretory mechanisms^11,12^. Shells grow in a linear fashion by adding new material to the growing edge; pigments are thus incorporated into the shell along this growing edge, and change their position continually as new shell is added^13^. Pigments may be laid down in the outer or other layers of mollusk shells^10,12^. To date, three common classes of pigments including melanin, carotenoids and tetrapyrroles was identified in mollusk^10^. Melanin is thought to be responsible for dark colors of mollusks^10^. The molecular pathway for the synthesis of melanin was considered important to contribute the shell (and pearl) pigmentation^14,15^, and the tyrosinase genes, the most important enzymes in the regulation and production of melanin has been identified in *Pinctada fucata*^14^, *Mizuhopecten yessoensis*^16^, *R. philippinarum*^17^, *Crassostrea gigas*^18^ and *Amusium pleuronectes*^19^*.* Addition, microphthalmia-associated transcription factor (MITF), a positively regulated gene of tyrosinase was also cloned in *Patinopecten yessoensis*^20^ and *Meretrix petechialis*^21^. Carotenoids is thought to be related to the yellow and orange pigment of the shells^22^. In *Hyriopsis cumingii*, the apolipoprotein (Apo) gene which related to carotene metabolism was found to be expressed differently in purple and white inner-shell color^23^. In *Patinopecten yessoensis,* GWAS identified three genes (*LDLR*, *FRIS*, and *FRIY*) involved in the carotenoid metabolism were responsible for the shell colors of this species^24^. In *Chlamys nobilis*, the SRB-like-3 gene which responsible for carotenoid deposition was believe associated with the orange color^25^. Transcriptome analysis of *Mercenaria mercenaria* showed the genes related to Carotenoid and Porphyrin and chlorophyll metabolism appeared to be associated with shell pigmentation^26^. Porphyrins were often associated with red, brown or purple shell coloration^27^. In *Calliostoma zizyphinum,* the genes associated with the synthesis of porphyrins were proved to participate in the synthesis of shell color^28^. Shell coloration has been shown to involve a complex bioprocess involving the original pigment and the chemical interactions between the pigments and the shell matrix^10^. To clarify the mechanism of shell color formation requires more research data. However, no miRNAs have yet been demonstrated to be involved in the determination of mollusk shell color. MicroRNAs (miRNAs) are a class of non-coding RNAs of approximately 20 nucleotides (nt) long that play important roles in regulating gene expression in biological processes^29^. miRNAs have also been suggested to play a crucial role in shell biomineralization. The miRNAs pm-miR-2386 and pm-miR-13b were predicted to participate in biomineralization in the pearl oyster *Pinctada martensii* by regulating the formation of the organic matrix or the differentiation of mineralogenic cells during shell formation^30^, while miR-2305 was also shown to participate in nacre formation in the shell^31^. These results suggest that miRNAs may play a role in the formation of shell color; however, no miRNAs have yet been demonstrated to be involved in the determination of mollusk shell color.

In this study, we constructed high-quality small-RNA transcriptomes for three shell-color lines of the Manila clam *Ruditapes philippinarum*, to provide basic data to further our understanding of the mechanisms regulating shell-color formation in this species.

## Results

### Sequence analysis of miRNAs

**Figure 1:**
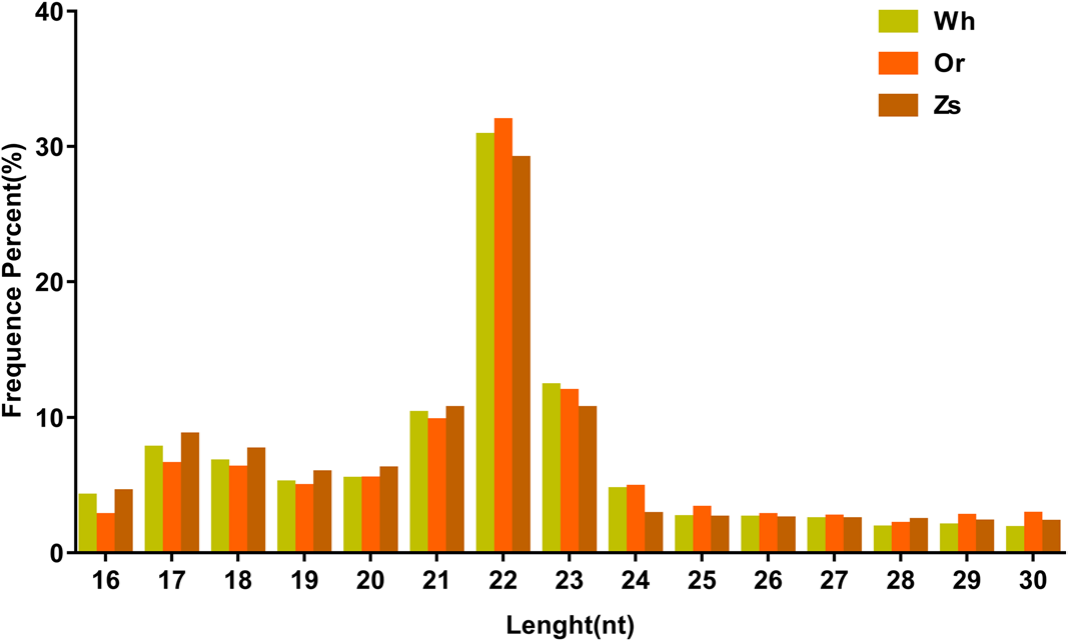
Length distribution of sequencing reads in three libraries.

To characterize small RNA transcripts, we constructed three small-RNA libraries for the three shell-color morphs of *R. philippinarum* using Illumina high-throughput sequencing technology. Totals of 22,042,778 (Wh), 22,357,681 (Or), and 21,442,934 (Zs) raw reads were obtained. After removing low-quality reads, adaptors, and small reads (length < 16), 3,891,284, 10,769,530, and 12,561,241 clean reads, respectively, were obtained, and 1,323,648, 16,671,16, and 1,140,082 unique sequences were obtained by clustering. The sequence lengths of the clean reads ranged from 16 to 30 nt, with most miRNAs being 21–23 nt long (Fig. 1).

### Differential expression of miRNAs in *R. philippinarum* with different shell colors

**Figure 2:**
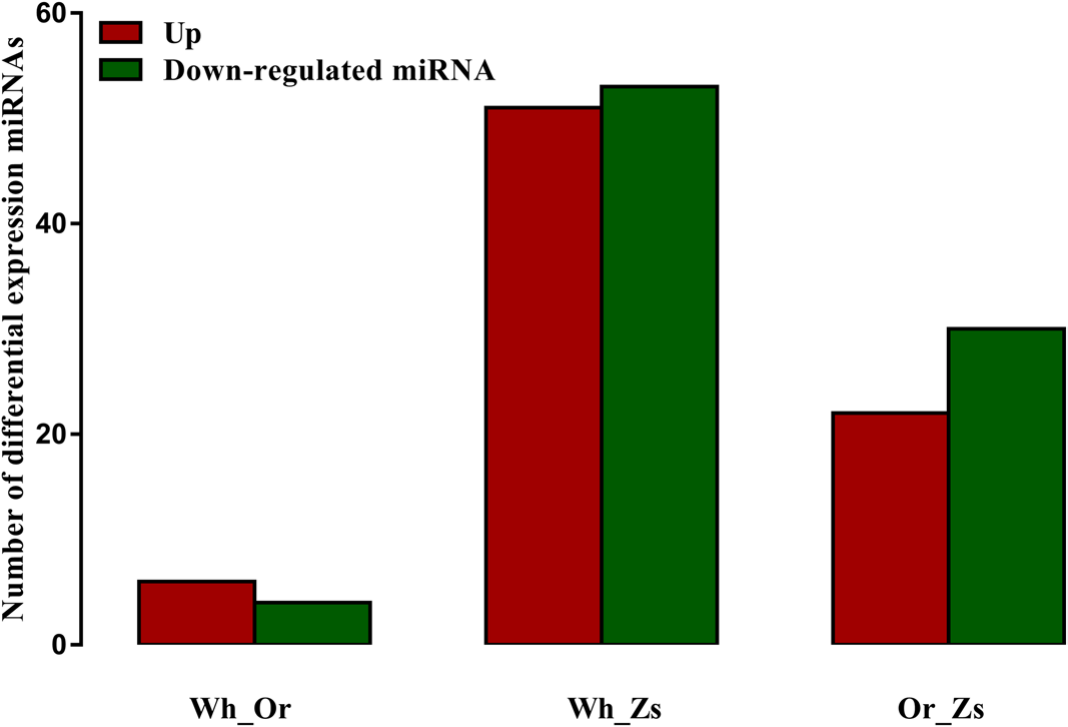
Differentially expressed miRNAs (DEMs) in three pairwise comparison groups. Red and green indicate up- and down-regulated miRNAs, respectively.

**Figure 3:**
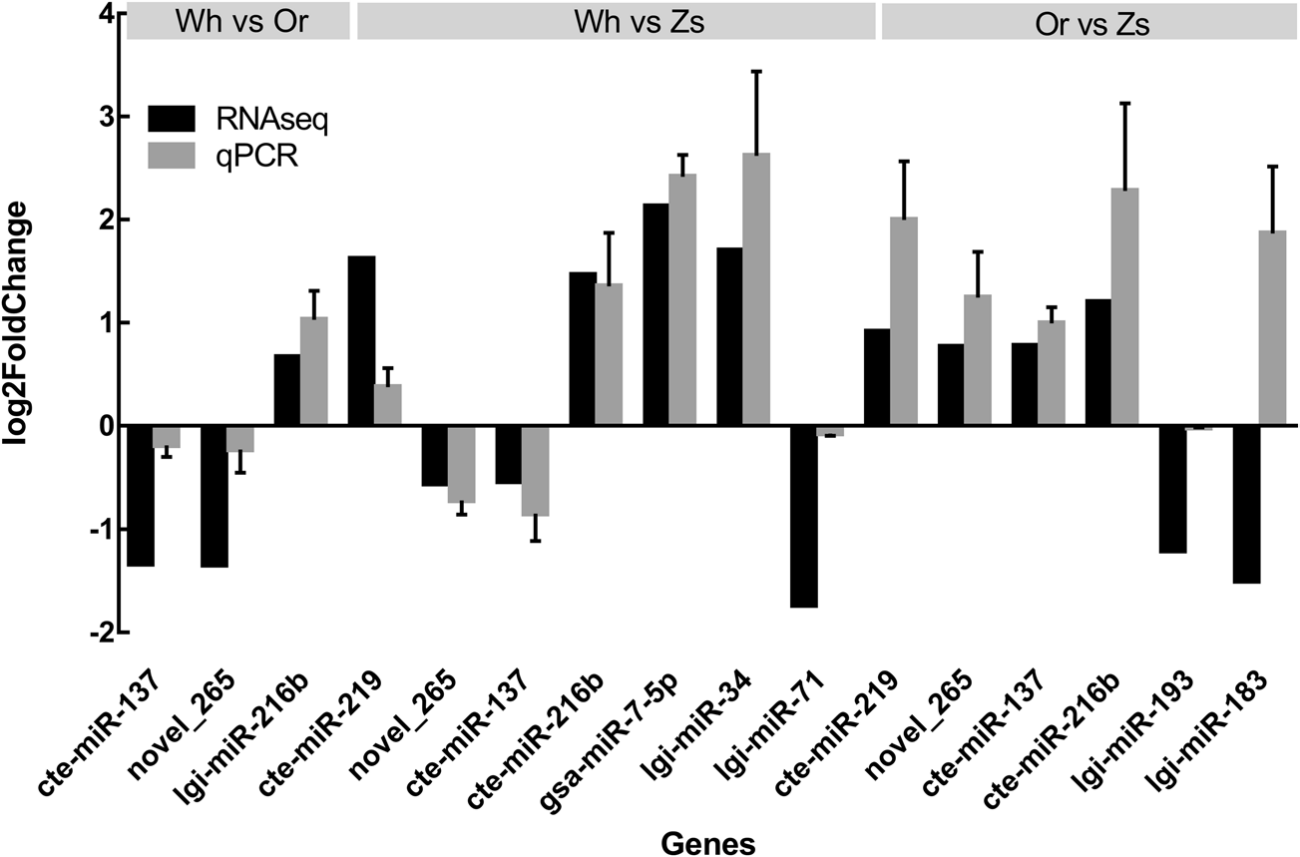
Expression of 10 selected miRNAs determined by qRT-PCR.

A total of 114 miRNAs were identified that were the same as or similar to known mature miRNAs (Supplementary Table S1), while 208 miRNAs were predicted to be novel miRNAs with hairpin structures (Supplementary Table S2). There were 10 significantly DEMs in Wh compared with Or (six upregulated and four downregulated), 104 significant DEMs in Wh compared with Zs (51 upregulated and 53 downregulated), and 52 significant DEMs in Or compared with Zs (22 upregulated and 30 downregulated) (Fig. 2; Supplementary Table S3). Moreover, only 2 known miRNAs including miR-137 and miR-216 were EEMs between in Wh and Or groups, there another 14 and 36 know DEMs between Or and Zs shell -color strains and between the Wh and Zs shell -color strains respectively. Several miRNAs previously identified as related to pigmentation synthesis, including MiR-137^32,33^ and miR-7-5p, were included among the DEMs^34^. The expression levels of ten selected miRNAs were validated by qRT-PCR, and all showed a consistent pattern with those from small-RNA sequencing analysis (Fig. 3), indicating reliability of the analysis.

### Target-gene prediction and functional analysis of DEMs

**Figure 4:**
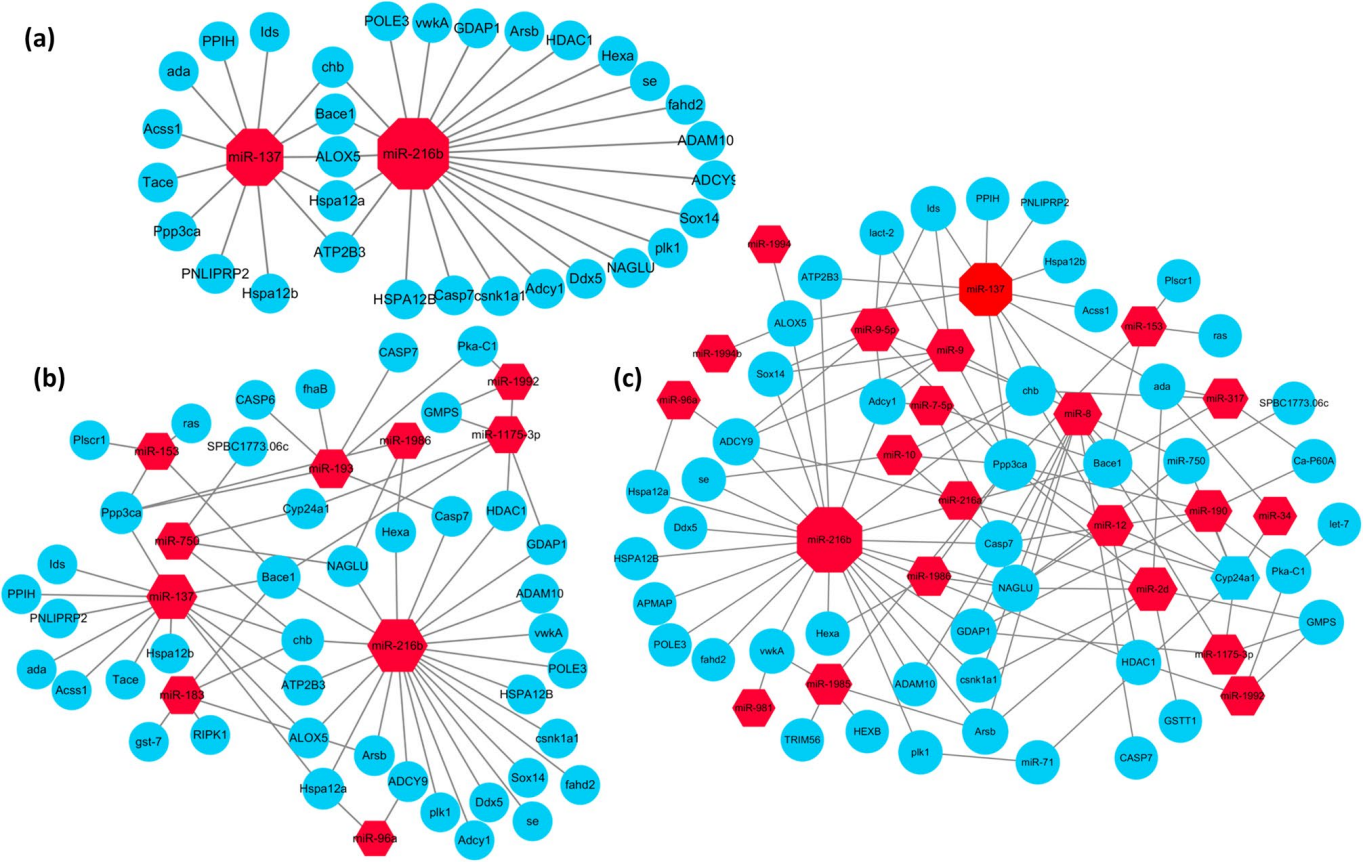
Potential regulatory networks between DEMs and their target genes. (**a**) Wh vs Or, (**b**) Wh vs Zs, (**c**) Or vs Zs. The regulation network of miRNAs and their target genes was illustrated by Cytoscape software. Red Hexagons and blue circles represent miRNAs and target genes, respectively.

The 10, 104, and 52 DEMs from the above three comparisons corresponded to 6228, 14,793, and 10,479 target genes, which were successfully mapped to existing gene categories and categorized into 66, 47, and 43 functional groups, respectively (Supplementary Table S4). KEGG pathway analysis was used to estimate the biochemical metabolic pathways and functions of the candidate target genes, and showed that these target genes could be classified into several pigmentation-related pathways, including the Wnt, Notch, and dopaminergic synapse signaling pathways (Supplementary Table S5). Illustration of the regulatory network between the DEMs and their targets (Fig. 4) indicated that miR-137 and miR-216b might play central roles in the interacting network. These results suggested a complicated regulatory network between miRNAs and their targets in pigmentation differentiation in *R. philippinarum*.

## Discussion

miRNAs represent a large class of small noncoding RNAs that bind to the 3′-untranslated region of target genes, thus affecting cleavage or translational repression to regulate gene expression at the posttranscriptional level. miRNAs play important roles in diverse biological processes, including cell proliferation, differentiation, and apoptosis. miRNAs have also recently been suggested to play a key role in the formation of body color in animals. For example, miR-137 was shown to influence the phenotype of coat color in transgenic mice by down-regulating microphthalmia-associated transcription factor (MITF)^33^, while miR-8 was required for the proper spatial patterning of pigment on the dorsal abdomen of the fruit fly^35^.

Several miRNAs have been reported to be involved in skin-color formation in fish. miR-429 silencing resulted in a visible change in skin pigmentation in the common carp (*Cyprinus carpio*)^36^, and miR-138-5p and miR-722 were predicted to play potential roles in skin-color differentiation in red tilapia^37^. However, reports on the functions of miRNAs in relation to shell color in mollusks remain scarce. In the present study, we compared the miRNAs from three shell-color strains of *R. philippinarum* and analyzed them using high-throughput sequencing technology, which identified a total of 322 miRNAs, including 114 known and 208 novel miRNAs. The numbers of DEMs differed significantly between clams with different shell colors.

It is worth noting that the expression levels of miR-137 and miR-216b differed significantly among the three shell-color strains, with miR-137 being significantly downregulated in Or compared with Wh and Zs. One of the miR-137 target gene- MITF mRNAs were previously reported to be mostly upregulated in the Or strain in *R. philippinarum*^38^. And the MITF target gene-tyrosinase gene were also found highest expression level in Or compare with Wh and Zs shell color strains of *R. philippinarum*^39^*.* These results suggest that miR-137 can also affect shell color in *R. philippinarum* by affecting the formation of melanin. In addition, only these two known miRNA shows different expression between the Or and Wh strains. There are more DEMs between Or and Zs, and between the Wh and Zs shell -color strains. Compared with the simpler shell color of the Or and Wh strains, the Zs strains exhibited more complex stripes and shell colors. This result is consistent with the transcriptome analysis in the four different shell color strains *R. philippinarum*^17^. These results indicate that more regulatory pathways are needed to regulate the formation of complex shell colors in this species. More studies were needed to confirm this conclusion. However, there have been no reports on the role of miR-216b in pigmentation to date.

Shell pigmentation is a complex and precise process. To obtain a better understanding of the mechanism of pigmentation in *R. philippinarum*, we predicted and analyzed the target genes of DEMs based on the clam’s genomic data. KEGG pathway analysis showed that DEM target genes including casein kinase I, glycogen synthase kinase 3, protein kinase A and Jun-N-terminal kinase were mainly involved in the Hedgehog and Wnt signaling pathways, which might be involved in pigment formation and regulation^40–43^. In Mollusca, Hedgehog signaling was shown to be involved in muscle differentiation in *Sepia officinali*s and in myogenesis in *Crassostrea gigas*^44,45^. The Hedgehog signaling pathway was also involved in the differentiation of retinal pigment epithelium cells in *Xenopus*^46^. Hedgehog signaling also plays a critical role in neural development^47^, including pattering of the neural tube in mice and chicks^48,49^. The neural system of the mantle has been suggested to be involved in pigmentation in mollusks by sensing the current pigmentation pattern and governing the formation of new pigment and shell material at a fine positional level^6^. The patterns of pigment on mollusk shells have been suggested to arise from the stimulation of secretory cells in the mantle by the animal’s central nervous system^50^. Shell pigmentation in *Haliotis asinine* was considered to be controlled by numerous cells and the secretion of pigments via tubule-based secretory tubules^12^. These results suggest a correlation between shell-color formation and nervous system development. The Wnt signaling pathway plays important roles in embryonic development and in adult tissue homeostasis and regeneration^51^. Wnt signaling was also shown to regulate pigment-cell differentiation in zebrafish^40^ and to promote the differentiation of neural crest cells into melanocytes in mice^52^. Wnt genes were also differentially expressed in mantle regions underlying shell with different pigmentation in the *Yesso scallop*^53^. We also previously found an expansion of Wnt genes and their potential roles in shell-color pattern in *R. philippinarum*^54^. Furthermore the relationship between Wnt/β-catenin signaling and MITF has been reported to be a critical feature in melanocyte development and subsequent pigmentation^55^. These results suggest that the Wnt signaling pathway might play a key role in pigmentation, especially in the formation of melanin, in *R. philippinarum*.

## Materials and methods

### Sample collection and RNA extraction

**Figure 5:**
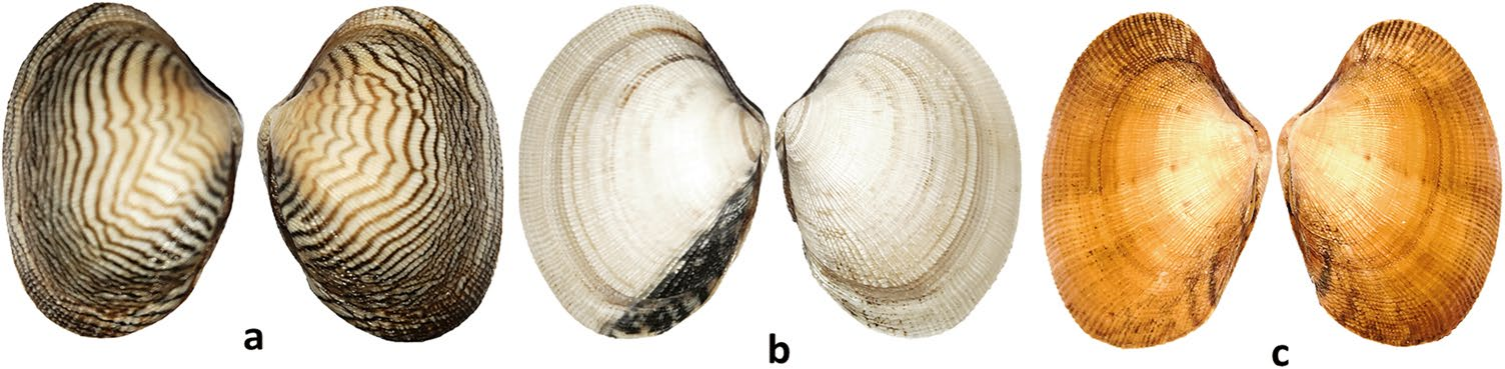
The three shell color lines of *R. philippinarum* used to construct small RNAs transcriptomes: zebra stripe (**a**), white (**b**), and orange (**c**).

Three shell-color lines of *R. philippinarum*, zebra stripe (Zs), orange (Or), and white (Wh) (Fig. 5), established by self-copulation for six generations, were collected from a farm in Dalian, China, and transported to the laboratory. Mantles were dissected from six clams from each strain, immediately ground into a powder in liquid nitrogen, and then stored at − 80 °C before processing for RNA extraction. Each sample was duplicated.

Total RNA was extracted from the mantle using TRIzol reagent (Invitrogen, Carlsbad, CA, USA) according to the manufacturer’s instructions. RNAs of 15–30 nt long were purified by 15% denaturing polyacrylamide gel electrophoresis, ligated to 3ʹ and 5ʹ adapters, and reverse transcribed using an Illumina sequencing kit (Illumina, San Diego, CA, USA). The polymerase chain reaction (PCR) products were purified using phenol/chloroform extraction and ethanol precipitation and sequenced by Beijing Biomarker Technologies (Beijing, China) using an Illumina HiSeq 2000 Genome Analyzer (Illumina).

### Small-RNA sequencing and read alignment

Raw sequence reads for the Wh, Or, and Zs strains were analyzed as described previously^56^. Briefly, reads with excessively small tags or from contaminating adapter-adapter ligation were removed. Clean reads were then aligned to the RepBase (v15, http://www.girinst.org), Rfam (http://www.sanger.ac.uk/Software/Rfam/ftp.shtml), and GenBank databases to remove small RNAs that mapped to annotated exons, repeats, rRNAs, tRNAs, or small nuclear RNAs. Unique sequences were obtained for further analysis.

### Identification of conserved and novel miRNAs

Clean reads were mapped to the clam genome^38^ using SOAP^57^ and their expression and distribution were analyzed. To identify conserved miRNA sequences, reads were aligned using miRBase (release 20.0) to search for perfectly matched short reads. Reads that did not match the databases were marked as unannotated, and unannotated sequences were aligned with the clam transcriptomic sequences to predict potential novel candidate miRNAs. The secondary structures of these candidate miRNAs were further analyzed using miREv and miRDeep2.0 software^58,59^.

### Differential expression analysis of miRNAs

We compared miRNA expression among the three shell-color groups by normalizing the read counts for each identified miRNA to the total number of reads in each given sample. Differential expression between two shell-color groups was analyzed using the DEGseq package, and the P-value was adjusted using the q-value. Fold-changes in miRNAs were calculated as the ratio of read counts in different shell-color groups followed by log2 transformation. The q value < 0.05 and log_2_ (fold-change) ≥ 1.0 were considered as the thresholds for significant differential expression.

### Validation of *R. philippinarum* miRNAs by stem-loop quantitative real-time-PCR (qRT-PCR)

Ten significantly differentially expressed miRNAs (DEMs) including miR-137 and miR-216b that expressed differed significantly among the three shell-color strains and other five known miRNAs and one putative novel miRNA were selected randomly for analysis by RT-PCR, with the U6 fragment as an internal control (Table 1). miRNA was extracted from mantle tissue using a miRcute microRNA isolation kit (Tiangen, China) and reverse transcribed using a miRcute miRNA first-strand cDNA kit (Tiangen). qRT-PCR was performed using a LightCycler 480 II Real-time PCR Instrument (Roche, Switzerland) with a miRcute miRNA qPCR Detection Kit (SYBR Green) (Tiangen). The reactions were carried out in a total volume of 20 μl, including 10 µl 2 × miRcute Plus miRNA PreMix, 0.4 µl each of sense and antisense primers (10 µM), 6.6 µl diethylpyrocarbonate-treated water, and 2 µl of DNA template according to the following steps: initial denaturation at 95 °C for 5 min, followed by 40 cycles of 95 °C for 15 s, 94 °C for 20 s, and 60 °C for 34 s. For every miRNA, qRT-PCR reactions for each sample were repeated three times. The relative expression levels were calculated using 2^−△△Ct^ method.

**Table 1:** Primers used in stem-loop qRT-PCR.

	Primer	Mature
cte-miR-137	GGCTCCAATAGCTTGAGAATACACGTAG	TATTGCTTGAGAATACACGTAG
lgi-miR-71	GCGTCCTGAAAGACAAGGGTAGTGAGATG	TGAAAGACAAGGGTAGTGAGATG
lgi-miR-34	GCTGGCAGTGTGGTTAGCTGGTAGT	TGGCAGTGTGGTTAGCTGGTAGT
gsa-miR-7-5p	GCGTCCTGGAAGACTTGTGATTGAGTTGTT	TGGAAGACTTGTGATTGAGTTGTT
cte-miR-219	GGCTGTATGTCCAAACGCAATTCT	TGATTGTCCAAACGCAATTCT
cte-miR-153	GGCGTCCTTGCATAGTGACAATAGTGATC	TTGCATAGTCACAAAAGTGATC
lgi-miR-193	GTACAGGCCTGCAAAATCCCAAC	TACTGGCCTGCAAAATCCCAAC
lgi-miR-183	GCTATGGCACTGGTAGAATTCACGG	AATGGCACTGGTAGAATTCACGG
novel_265	GGCCGCTAAATGCTTGAGAATACACGT	TTATTGCTTGAGAATACACGT
cte-miR-216b	GCGCGTCCTAATATCAGCTGGTAATTCTGA	TAATATCAGCTGGTAATTCTGA
U6	ATTGGAACGATACAGAGAAGATTAG	

### Target gene prediction and analysis of potential mRNA–regulatory miRNA networks involved in shell-color formation

Target genes were predicted using the computational prediction programs RNAhybrid^60^, Miranda^61^, and TargetScan^62^. Genes supported by any two algorithms were considered to be likely potential miRNA targets. The target reference sequences were genome unigenes and annotation information for *R. philippinarum*. We further investigated the functions of the miRNAs in *R. philippinarum* pigmentation by enrichment analysis of the predicted target genes by Gene Ontology (GO) (http://www.geneontology.org/) and Kyoto Encyclopedia of Genes and Genomes (KEGG) pathway (http://www.genome.jp/kegg/) analyses. To identify key DEMs and their regulatory networks in shell-color formation, the predicted mRNA–miRNA regulatory interactions between the DEMs and their target genes in the top 10 pathways were visualized using Cytoscape version 3.6.1^63^.

## Supplementary Information

Supplementary Information 1.

Supplementary Information 2.

Supplementary Information 3.

Supplementary Information 4.

Supplementary Information 5.

Supplementary Information 6.

## Data Availability

All the sequencing reads were deposited in the NCBI Short Read Archive database (http://www.ncbi.nlm.nih.gov/sra/) and are retrievable under the accession numbers SAMN10337891, SAMN10337892, SAMN10337893, SAMN10337894, SAMN10337895, and SAMN10337896. The *R. philippinarum* assembly was deposited with the accession number QUSP00000000 under PRJNA479743. All sequence data for the *R. philippinarum* genome were deposited in the NCBI Sequence Read Archive under the accession numbers SRR7716263–SRR7716297.
