# Supplementary material for: Identification of shell-color-related microRNAs in the Manila clam Ruditapes philippinarum using high-throughput sequencing of small RNA transcriptomes
Source: Sci Rep. 2021 Apr 13;11:8044. doi: 10.1038/s41598-021-86727-9 (PMC8044141; doi:10.1038/s41598-021-86727-9)
Supplement: Supplementary file 6 — Supplementary Information 6. [file 41598_2021_86727_MOESM6_ESM.docx]

Supplementary Table 1. The known mature miRNAs

Supplementary Table 2. The novel miRNAs

Supplementary Table 3.The differentially expressed miRNAs (DEMs) in three pairwise comparison groups

Supplementary Table 4 .GO categories of the predicted target genes of DEMs

Supplementary Table 5 .KEGG enrichment of the predicted target genes of DEMs
